# The contribution of apixaban renal clearance to total clearance

**DOI:** 10.1007/s11239-015-1220-8

**Published:** 2015-05-12

**Authors:** Charles Frost, Rebecca A. Boyd

**Affiliations:** Bristol-Myers Squibb, Princeton, NJ USA; Pfizer Inc, Groton, CT USA

To the Editor,

Kreutz [1] reported that the renal clearance of absorbed active apixaban was 50 %. From the references cited, it is assumed that this value was obtained using the reported absolute oral bioavailability of 50 % [2] and data from a human absorption, distribution, metabolism, excretion (ADME) study, in which ~23 % of the radiolabeled dose was recovered unchanged in urine [3]. We would like to bring to the author’s attention the error in the percentage of the orally available apixaban eliminated by renal clearance, as reported in Table 1 [1].

Raghavan et al. [3] describes the disposition of apixaban following oral administration of [C^14^]apixaban solution in ten healthy male subjects. The data described in this study provide important information regarding the disposition of apixaban in humans; however, these data were not used to determine the contribution of apixaban renal clearance to total clearance. The purpose of this study was to identify the routes and extent of apixaban elimination, as well as to assess the metabolism of apixaban and qualitatively identify its metabolites. The results of this study were presented as the percent of the total radioactive dose recovered as apixaban (unchanged drug) and its metabolites, and demonstrated that apixaban was eliminated renally as well as through metabolism and excretion into the intestinal tract. While this study identified renal excretion as one pathway of apixaban elimination, it is not possible to determine its contribution to the total clearance of apixaban from these data. This would require quantitatively differentiating the fraction of the dose that was not absorbed from the fraction of the dose that was absorbed and excreted unchanged directly into the intestinal tract, i.e. knowing the absolute bioavailability of apixaban in these subjects.

Because apixaban is an orally administered drug with multiple routes of elimination, the only appropriate method for determining the contribution of apixaban renal clearance (CLR) to total clearance (CLT) is to determine clearance after intravenous administration. This approach to calculating the percent contribution of CLR to CLT (% CLR) represents the “gold standard”, as the absolute bioavailability of the intravenous dose is 100 %. Apixaban total clearance and renal clearance have been determined following intravenous administration in 2 clinical pharmacology studies [4, 5]. In these studies, apixaban CLT was calculated using the apixaban dose and the area under the apixaban plasma concentration time curve from time 0 to infinity [AUC_(INF)_], as follows:

$$ {\text{CLT }} = {\text{ Dose}}/{\text{AUC}}_{{ ( {\text{INF)}}}} $$

Apixaban CLR was calculated using the total urinary recovery of apixaban (URT) and the area under the apixaban plasma concentration time curve from time 0 to T determined over the same time interval (T), as follows:

$$ {\text{CLR }} = {\text{ URT}}/{\text{AUC}}_{{(0 - {\text{T}})}} $$

In the first study, a total of 30 subjects received apixaban in 1 of 5 dose panels (0.5–5 mg) (6 subjects per panel). The results of this study demonstrated that renal clearance represented approximately 17–30 % of the apixaban total clearance. Apixaban was also administered intravenously in a phase I drug–drug interaction study with rifampin (*n* = 20) [5]. In this study, the contribution of apixaban renal clearance to total clearance was 34 %. The average contribution of renal clearance across these two studies was approximately 27 %, supporting the statement in both the Eliquis^®^ (apixaban) SmPC and USPI [2, 6]. If the author’s intent is to compare the contribution of renal excretion to elimination across the compounds, then the contribution of renal clearance to total clearance, as presented above, is the most appropriate value for that comparison.

We hope that these additional data help clarify the apixaban pharmacokinetic profile, specifically with regard to apixaban disposition and the contribution of renal excretion to overall elimination.
